# A Non-Hydrolytic Sol–Gel Route to Organic-Inorganic Hybrid Polymers: Linearly Expanded Silica and Silsesquioxanes

**DOI:** 10.3390/gels9040291

**Published:** 2023-04-02

**Authors:** Katrin Krupinski, Jörg Wagler, Erica Brendler, Edwin Kroke

**Affiliations:** 1 Institute of Inorganic Chemistry, Department of Chemistry and Physics, Technische Universität Bergakademie Freiberg (TUBAF), Leipziger Strasse 29, 09596 Freiberg, Saxony, Germany; katrinlippe@gmx.net (K.K.); joerg.wagler@chemie.tu-freiberg.de (J.W.); 2 Center of Efficient High Temperature Processes and Material Conversion (ZeHS), Technische Universität Bergakademie Freiberg (TUBAF), Winklerstr. 5, 09599 Freiberg, Saxony, Germany; 3 Institute of Analytical Chemistry, Department of Chemistry and Physics, Technische Universität Bergakademie Freiberg (TUBAF), Leipziger Strasse 29, 09596 Freiberg, Saxony, Germany; erica.brendler@chemie.tu-freiberg.de

**Keywords:** inorganic/organic networks, non-aqueous gels, arylene bridged, chlorosilanes, pyridine, ^29^Si solid state NMR

## Abstract

Condensation reactions of chlorosilanes (SiCl_4_ and CH_3_SiCl_3_) and bis(trimethylsilyl)ethers of rigid, quasi-linear diols (CH_3_)_3_SiO–*AR*–OSi(CH_3_)_3_ (*AR* = 4,4′-biphenylene (**1**) and 2,6-naphthylene (**2**)), with release of (CH_3_)_3_SiCl as a volatile byproduct, afforded novel hybrid materials that feature Si–O–C bridges. The precursors **1** and **2** were characterized using FTIR and multinuclear (^1^H, ^13^C, ^29^Si) NMR spectroscopy as well as single-crystal X-ray diffraction analysis in case of **2**. Pyridine-catalyzed and non-catalyzed transformations were performed in THF at room temperature and at 60 °C. In most cases, soluble oligomers were obtained. The progress of these transsilylations was monitored in solution with ^29^Si NMR spectroscopy. Pyridine-catalyzed reactions with CH_3_SiCl_3_ proceeded until complete substitution of all chlorine atoms; however, no gelation or precipitation was found. In case of pyridine-catalyzed reactions of **1** and **2** with SiCl_4_, a Sol–Gel transition was observed. Ageing and syneresis yielded xerogels **1A** and **2A**, which exhibited large linear shrinkage of 57–59% and consequently low BET surface area of 10 m^2^⋅g^−1^. The xerogels were analyzed using powder-XRD, solid state ^29^Si NMR and FTIR spectroscopy, SEM/EDX, elemental analysis, and thermal gravimetric analysis. The SiCl_4_-derived amorphous xerogels consist of hydrolytically sensitive three-dimensional networks of SiO_4_-units linked by the arylene groups. The non-hydrolytic approach to hybrid materials may be applied to other silylated precursors, if the reactivity of the corresponding chlorine compound is sufficient.

## 1. Introduction 

Organic-inorganic hybrid materials usually have properties intermediate between organic polymers and inorganic substances, such as oxide glasses or ceramics [1,2,3]. A well-known approach to organic-inorganic hybrid materials uses the classic Sol–Gel process based on hydrolysis reactions of (semi)metal alkoxides [4,5,6,7]. Non-aqueous or non-hydrolytic [8,9,10,11] and non-oxide [12,13] Sol–Gel systems have also been reported. The so-called non-hydrolytic routes frequently refer to oxidic products, which may be used for similar applications as oxide products derived from hydrolytic routes; these include catalyst supports [14], optical materials [15], or aerogels [16,17,18], among many others [4,5,6,7,8,9].

**Scheme 1:**
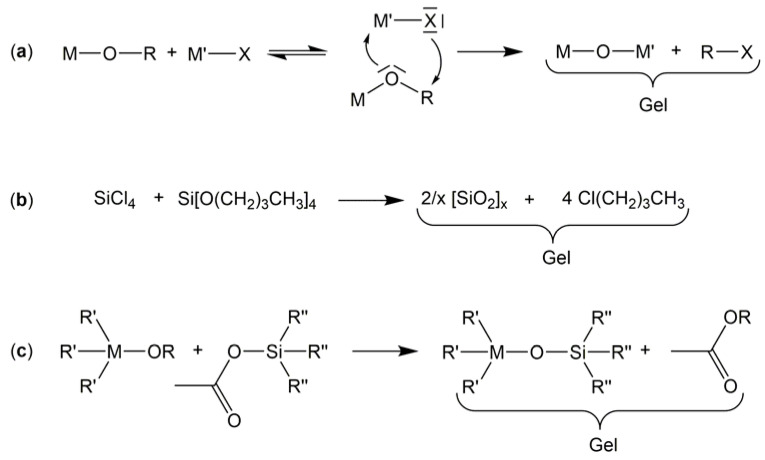
Examples of non-hydrolytic oxide-based Sol–Gel strategies: (**a**) Generic reaction of metal alkoxide and metal halide moiety; (**b**) Reaction of silicon tetrachloride and tetra-*n*-butoxysilane; (**c**) Generic reaction of a metal alkoxide and an acetoxysilane.

Non-hydrolytic Sol–Gel systems are frequently based on metal halides and their reactions with oxygen containing organic or organometallic compounds such as alkoxides or acetoxides. They yield polymeric networks that are comprised of M–O–M’ backbone units. Thus, these non-aqueous systems are basically alternatives to the classic aqueous Sol–Gel routes as they afford oxides or metal oxide based organic-inorganic hybrid materials. The M–O–M’ bridges are generated via a nucleophilic attack of the oxygen atoms at the metal atom of the halide. Therefore, the common principle of most non-hydrolytic Sol–Gel processes relies on a scission of carbon-oxygen-bonds instead of hydrolysis involving a nucleophilic attack of OH-groups (Scheme 1a). The (semi)metals M and M’ may be identical. For example, the reaction of SiCl_4_ with tetra-*n*-butoxysilane (Scheme 1b) has been used to generate silica gels upon formation of the liquid side product *n*-butylchloride [19]. The underlying reaction may be considered as an ether cleavage. This route can also be applied for other alkyl groups and may be simplified by using alcohols like methanol, ethanol, or isopropanol in order to form the alkoxide in situ via reactions with an element halide. In analogy to silica gels [20], other single component oxide gels (M = M’), such as alumina [21], titania [22] gels, or tungsten oxide [23], were prepared. Different mixed oxide ceramics such as TiO_2_/SiO_2_ [24], TiO_2_/Al_2_O_3_ [25], SiO_2_/Al_2_O_3_, or SiO_2_/ZrO_2_ [24,26] were synthesized using the same approach. This route has also been applied to prepare multinary solids such as YAG (yttrium aluminum garnet) powders [27], cordierites [28], or β-SiAlON:Eu powders [29]. In many cases, precipitation of the products, rather than Sol–Gel transition and monolith formation, has been reported. A number of related approaches, such as nonhydrolytic solvothermal synthesis of oxide nanoparticles under anhydrous conditions, e.g., [30], have been reported in recent years. In addition, numerous reports on the controlled aggregation and gelation of (non-hydrolytically) formed oxide colloids, i.e., nano-particle dispersions or sols, have been published [31].

Pronounced Lewis acidic metal halides, such as TiCl_4_, react with organic ethers, e.g., diisopropyl ether or THF (tetrahydrofuran), to give mesoporous TiO_2_-carbon nanocomposites [32]. In this process, which involves no additional solvent, the ether acts as both an oxygen donor and source of oxide and as the sole carbon source.

Acetoxysilanes and metal alkoxides (the latter in analogy to metal halides) react with formations of carboxylic acid esters and of non-aqueous oxide gels (Scheme 1c). The alkoxide may be a compound of the main group elements (silicon [33] and aluminum [21,33]) or of a transition metal (zirconium [26,33] or titanium [33,34]).

Further non-hydrolytic Sol–Gel systems are based on reactive organometallic compounds or alkoxides and further organic oxygen-containing compounds, e.g., acetone and zinc alkoxides react with formation of gels under enolization of the ketone and elimination of the corresponding alcohol [35]. This reaction behavior is observed only for non-acidic alkoxides.

Only few non-oxide Sol–Gel systems are known, i.e., Sol–Gel routes to sulfides, carbides, or nitrides, which lead to monolithic xerogels [12,13]. Some are related to the synthesis described here. In analogy to the hydrolysis and condensation reactions of the classic oxide Sol–Gel systems, compounds of the heavier chalcogenides may be considered as precursors [12,36].

**Scheme 2:**
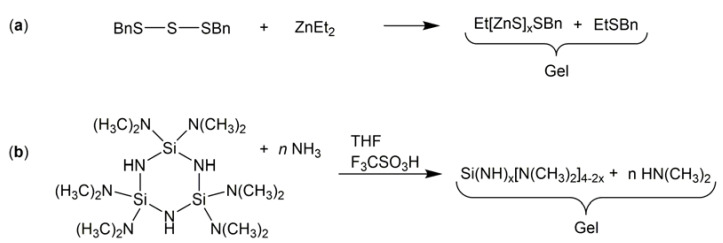
Examples of non-hydrolytic non-oxide-based Sol–Gel strategies: (**a**) Reaction of dibenzyltrisulfide and diethylzinc; (**b**) Reaction of a dimethylamino substituted cyclotrisilazane and ammonia.

Many papers on Sol–Gel synthesis routes to sulfides were published, but only a few Sol–Gel transitions were reported. For ZnS it was described that treatment of Et_2_Zn or [Zn(SR)_2_]_x_ with H_2_S generates precipitates, while reactions of dibenzyl trisulfide (BnS)_2_S and Et_2_Zn in pentane (Scheme 2a) yielded transparent sols, gels, and xerogels after removal of the solvent [37]. The successful preparation of colloidal CdS and CdSe [38] gels, as well as CdSe sols obtained from Cd(ethoxyacetate)_2_ and Se(Si(CH_3_)_3_)_2_ [39], represents another non-oxide Sol–Gel strategy. CdS/CdSe aerogels can also be obtained from nanocrstyalline starting materials [40]. In another example, Ge(OEt)_4_ was treated with H_2_S to form sulfide gels [41]. Interesting nanostructured sulfide materials have been obtained from molybdenum chloride and S(Si(CH_3_)_3_)_2_ in chloroform [42]. A similar approach, that is also based on the formation of trimethylchlorosilane, has been used to generate polydimethylsiloxane (PDMS) analogous silicon-sulfur polymers [43].

Even more work has been published on the synthesis of nitride materials via Sol–Gel routes. It may be assumed that simple replacement of water by ammonia or amines leads to nitride gels. However, it turns out that, in most cases, this approach is not successful, probably due to the much higher basicity of NH_3_ and RNH_2_ when compared to H_2_O. The first example of a Sol–Gel process based on an ammonolysis reaction yielding Si/(C)/N gels starts from a dialkylamino substituted cyclic trisilazane (Scheme 2b); it can be described as a transamination reaction [44]. These so-called silicon diimide gels have subsequently been used for stationary phases in thin layer chromatography for organic acids [45].

Hexagonal boron nitride (h-BN) and ternary B/C/N ceramics have been synthesized using “silazanolysis” reactions of B-trichloroborazene and its alkyl substituted derivatives with hexa- and heptamethyl disilazane [46]. A three-dimensional network infiltrated with liquid (CH_3_)_3_SiCl is formed that is analogous to the oxide polymer networks infiltrated by solvents and liquid reaction products. The dried xerogels showed low specific surface areas (<35 m^2^⋅g^−1^) when compared to oxide xerogels. Pyrolysis at 1200 °C produced hexagonal boron nitride.

**Scheme 3:**
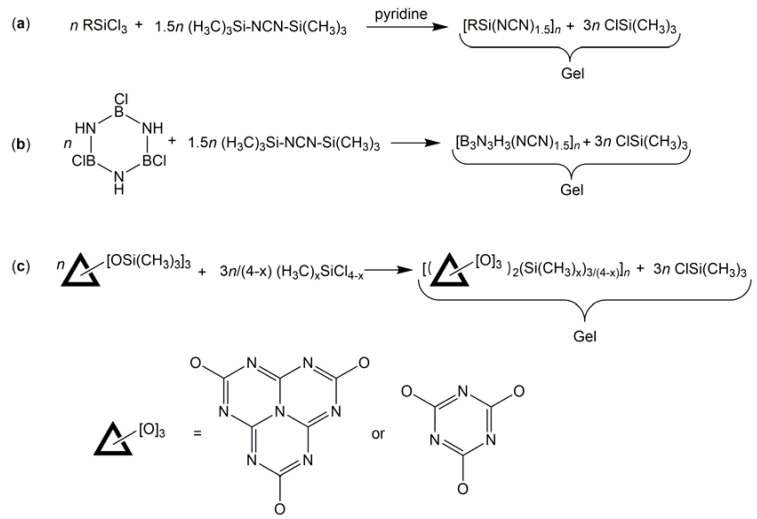
Examples of non-hydrolytic Sol–Gel strategies with release of (CH_3_)_3_SiCl as condensation product: (**a**) Generic reaction of trichlorosilanes and bis(trimethylsilyl)carbodiimide (R =, e.g., alkyl, aryl); (**b**) Reaction of B-trichloroborazene and bis(trimethylsilyl)carbodiimide; (**c**) Generic reaction of methylchlorosilanes and three-fold silylated cyameluric or cyanuric acid.

Another approach to synthesize gel precursors for carbide and nitride materials is based on the pseudo-chalcogen concept [47]. In principle, the oxygen atoms of polysiloxanes are replaced by carbodiimide (–N=C=N–) groups in order to obtain (poly)silylcarbodiimide compounds [48]. Reactions of bis(trimethylsilyl)carbodiimide (CH_3_)_3_Si–NCN–Si(CH_3_)_3_ and dichlorosilanes (such as (CH_3_)_2_SiCl_2_) give linear and cyclic oligomers [49], while reactions of this carbodiimide with trichlorosilanes (*R*SiCl_3_ with *R* = alkyl, aryl or H) or with tetrachlorosilane may result in the formation of transparent gels (Scheme 3a) [50,51]. Several attempts to extend this Sol–Gel system through replacing the chlorosilanes with other element halides were not successful. For example, analogous reactions with titanium chlorides, such as TiCl_4_ or CpTiCl_3_, led to the formation of precipitates; the use of RAlCl_2_ and gallium chlorides as starting materials gave soluble oligomers [52]. However, B-trichloroborazene (B_3_N_3_H_3_Cl_3_) reacts similarly to the chlorosilanes with bis(trimethylsilyl)carbodiimide and forms B/C/N gels (Scheme 3b) that can be transformed into B/C/N and B_4_C ceramics [53].

We modified the above-described Sol–Gel approach based on (CH_3_)_3_SiCl formation by replacing bis(trimethylsilyl)carbodiimide with trimethylsilyl esters of cyanuric and cyameluric acid (Scheme 3c). Their reactions with SiCl_4_ and CH_3_SiCl_3_ yielded networks consisting of three-fold bridged SiO_4_ and CH_3_SiO_3_ units [54]. Similar three-fold bridged networks were formed upon acid catalyzed reactions of trihydroxybenzene and tetraethoxysilane (TEOS) [55]. The same approach was used with TEOS and dihydroxybenzenes as bifunctional precursors [56].

Here we present the extension of the concept of reaction of element chlorides and silylated precursors to obtain “bridged” or “linearly expanded” silicas and silsesquioxanes. Reactions of rigid, quasi-linear bis(trimethylsilyl)ethers of diols of the type (CH_3_)_3_SiO–*AR*–OSi(CH_3_)_3_ (*AR* = quasi-linear arylene spacer) and chlorosilanes (SiCl_4_ and CH_3_SiCl_3_) afforded novel hybrid oligomers, polymers, and xerogels; the latter were investigated with X-ray diffraction (XRD), ^29^Si nuclear magnetic resonance (NMR), and Fourier-transform infrared(FTIR) spectroscopy, as well as with scanning electron microcopy coupled with energy-dispersive X-ray spectroscopy (SEM/EDX), elemental analysis, thermal gravimetry with differential thermal analysis (TG/DTA), and TG-FTIR.

## 2. Results and Discussion

### 2.1. Selection, Synthesis and Characterisation of the Starting Materials

**Figure 1:**
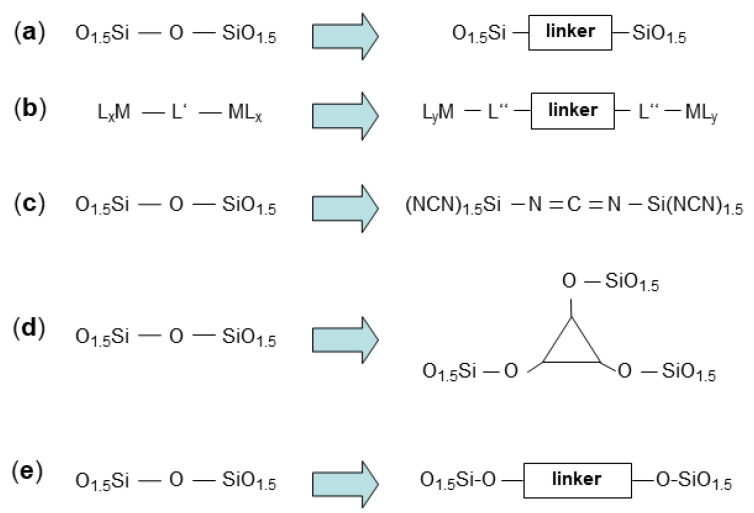
Approaches to “expanded oxides”: (**a**) The most frequently prepared hybrid materials which may be described as organically bridged silsesquioxanes; (**b**) MOFs (metal organic frameworks) can be considered as “organically expanded” coordination centers; (**c**) Replacing the oxygen atoms in Si–O compounds by carbodiimide units results in non-oxides, which are “expanded” compared to the oxide analogues; (**d**) “Trigonally expanded silicas” as prepared earlier; (**e**) “Linearly expanded silicas” as presented here.

As mentioned in the introduction, the goal of the present work was to synthesize “linearly expanded silica and silsesquioxanes”. The latter term defines hybrid materials that differ from the well-known “organically bridged” silsesquioxanes O_1.5_Si–[linker]–SiO_1.5_ [57,58,59,60] (Figure 1a), which feature various organic groups as linkers, and related systems such as polysilsesquioxanes [*R*–SiO_1.5_]_n_, with *R* being a terminal group (such as hydrocarbyl) [61,62]. These materials are based on the replacement of Si–O bonds by Si–C bonds. The present approach retains original Si–O motifs but alters the –O– bridge into an extended –O–[linker]–O– quasi-linear bidentate connector. Thus, these systems may be compared to metal organic frameworks (MOFs) [63], which may be considered “expanded” coordination centers (such as metal oxide centers) bridged by rigid bidentate ligands (such as terephthalate [O_2_C–C_6_H_4_–CO_2_]^2−^) symbolized as L_y_M–L″–[linker]–L″–ML_y_ (Figure 1b). This route to usually crystalline network structures has been extended to covalent organic frameworks (COFs) [64].

Silylcarbodiimides may also by viewed as “linearly expanded Si–O” compounds, since the oxygen atoms are replaced by NCN-units as alternative difunctional linkers of similar electronegativity [48,49,50,51,52,53]. This approach differs from the above oxides since Si–O bonds are replaced by Si–N bonds (Figure 1c). In the present work, we aimed to “expand” SiO_x_-units with rigid spacers without changing the Si–O bonds, i.e., incorporating Si–O–C linkages. In a former study, we used three-fold functional units as organic bridges (Figure 1d) [54]. The obtained hybrid materials are highly cross-linked gels. In order to synthesize polymeric gels with a lower degree of cross-linking, we decided to use two-fold functionalized rigid bridges (Figure 1e).

All attempts to generate the target materials using reactions of commercially available diols, including 4,4′-dihydroxybiphenyl with alkoxysilanes such as TEOS or tetramethoxysilane (TMOS), were not successful. The poor solubility of these diols in polar and non-polar organic solvents may be one of the reasons. In order to start from homogeneous solutions, we decided to react silylated diols with chlorosilanes.

4,4′-Bis(trimethylsiloxy)biphenyl **1** was synthesized according to a previously reported protocol [65]; the silylated product was additionally purified by crystallization from *n*-hexane. 2,6-Bis(trimethylsiloxy)naphthalene **2** was prepared analogously by silylation of the diol with hexamethyldisilazane (HMDS) using KOtBu as a catalyst. Upon vacuum distillation, compound **2** solidified to afford a colorless crystalline solid. Both starting materials (**1** and **2**) showed sharp melting points and delivered satisfying elemental analyses. In the FTIR spectra, the expected absorption bands for C_alkyl_–H, C_aryl_–H, C=C, Si–O, and Si(CH_3_)_3_ groups were present. For both compounds, the ^1^H, ^13^C, and ^29^Si NMR spectra exclusively exhibited the respective set of signals to be expected for the symmetric two-fold silylated products. No indications for monosilylated diols or for any other impurities were found.

**Figure 2:**
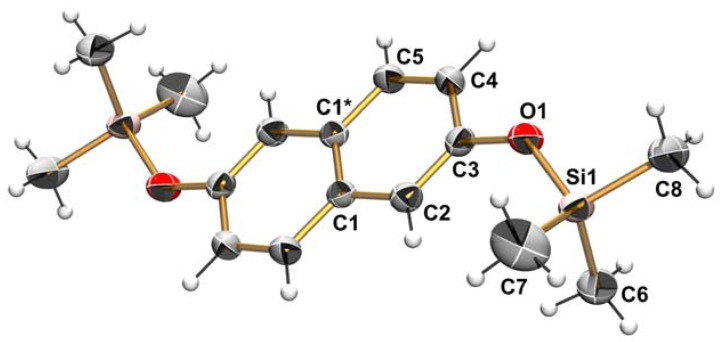
Molecular structure of **2** in the crystal (thermal displacement ellipsoids plotted at the 50% probability level). The bond C1–C1* of the molecule is located on a crystallographic center of inversion, the non-hydrogen atoms of the asymmetric unit are labeled, the asterisk * indicates a symmetry equivalent position. For clarity reasons hydrogen atoms (white balls) are not labeled.

In case of compound **2**, a single-crystal X-ray diffraction analysis was performed, providing complete structural information for this molecule (Figure 2). Selected bond lengths and angles are summarized in Table 1. All remaining structural data can be found in Table S1 (bond lengths), Table S2 (bond angles), and Table S3 (torsion angles).

The bonds between C4 and C5 (1.364 Å) and between C3 and C2 (1.364 Å) are significantly shorter than the remaining C–C bonds of the naphthalene group. Thus, four of the eleven bonds have an enhanced double bonding character. Nevertheless, all bond angles of the naphthalene unit are very close to 120° in accordance with a planar six-membered arene motif.

### 2.2. Oligomer and Polymer Formation in Solution

Solutions of the precursors (i.e., silylated diol **1** or **2**, chlorosilane CH_3_SiCl_3_, or SiCl_4_—with and without addition of pyridine, in THF as the solvent) were allowed to react at room temperature and at 60 °C. Only the reactions with SiCl_4_ and pyridine (**1aPy** and **2aPy**) afforded gels. All other reaction mixtures (**1a** = **1**/SiCl_4_/THF; **2a** = **2**/SiCl_4_/THF; **1b** = **1**/CH_3_SiCl_3_/THF; **2b** = **2**/CH_3_SiCl_3_/THF; **1bPy** = **1**/CH_3_SiCl_3_/pyridine/THF; **2bPy**= **2**/CH_3_SiCl_3_/pyridine/THF) were sealed in NMR tubes and investigated with ^29^Si NMR spectroscopy (using external D_2_O). The ^29^Si NMR spectrum of reaction mixture **1b** after one day at room temperature indicates a rather slow reaction; the spectrum is dominated by the signals of the starting materials, but the emergence of new signals at 31 ppm ((CH_3_)_3_SiCl) and −11 ppm (CH_3_SiCl_2_(O-aryl) confirm the transsilylation reaction even at room temperature and in absence of pyridine catalyst (cf. Figure S1 in the supporting information).

Upon further storage of reaction mixture **1b** (biphenyl precursor **1** and CH_3_SiCl_3_ in THF without pyridine) at room temperature, a new signal at −33 ppm (MeSiCl(O-aryl)_2_ moieties) emerges in the ^29^Si NMR spectrum; this is the sole product signal after 30 days (cf. Figure S2 in the supporting information). An analogous reaction mixture **2b** (using the naphthalene precursor **2** and CH_3_SiCl_3_ in THF without pyridine) exhibits similar behavior, but the emergence of the signal of CH_3_SiCl(O-aryl)_2_ groups (at −32 ppm) within one day and the disappearance of the signal of CH_3_SiCl_2_(O-aryl) groups (at −11 ppm) within 8 days indicates more rapid reaction of precursor **2** vs. **1** (cf. Figure S3 in the supporting information). Both systems have in common that transsilylation essentially stopped at the CH_3_SiCl(O-aryl)_2_ stage.

However, supplementing a reaction mixture such as **2b** with pyridine as a nucleophilic catalyst (**2bPy** = **2** plus CH_3_SiCl_3_ and pyridine in THF) enhances the already known transsilylation steps and gives rise to the emergence of a new ^29^Si NMR signal at −52 ppm (CH_3_Si(O-aryl)_3_ moieties) within 9 hours; this is the exclusive high field signal after 6 days (cf. Figure S4 in the supporting information). Thus, with pyridine, a complete substitution of all three Cl atoms of CH_3_SiCl_3_ by O-atoms occurs, while the reaction seems to cease at the stage of a two-fold substitution without addition of pyridine. This rate-increasing effect of pyridine is observed in a similar way for the biphenyl derivative (**1bPy**).

For tetrachlorosilane, the reaction progress of non-gel-forming reaction mixtures can also be followed in solution by ^29^Si NMR spectroscopy. The corresponding spectra are depicted in the supplementary material (Figures S5 and S6). They also indicate that the naphthalene derivative **2** reacts faster than the biphenyl compound **1**.

In the reaction mixtures of SiCl_4_ with **1** and **2** plus pyridine (**1aPy** and **2aPy**), a complete chlorine substitution is observed as in the reactions of CH_3_SiCl_3_ with **1** and **2** plus pyridine (**1bPy** und **2bPy**), i.e., formation of “SiO_4_“ and “CH_3_SiO_3_” and acceleration of the reactions. In addition, gelation also occurred. This indicates that the degree of cross-linking in the case of starting material CH_3_SiCl_3_ is not sufficient to form a rigid network. The silsesquioxane units obviously lead to fewer cross-linked and THF soluble oligomeric structures.

### 2.3. Gel Synthesis and Characterisation

The reaction mixtures of both silylated precursors **1** and **2** with SiCl_4_ and pyridine (**1aPy** and **2aPy**) formed gels within 24 hours. Over several days, strong syneresis was observed. After three weeks, the gels were dried in vacuum. The obtained xerogels **1A** and **2A** were brittle and glass-like; **1A** was white and **2A** was slightly brownish. Both xerogels were analyzed by elemental analysis, powder X-ray diffraction, and IR and solid state ^29^Si NMR spectroscopy. The latter spectroscopic investigations were also used to characterize the hydrolytic sensitivity of xerogels **1A** and **2A**.

EDX studies (performed during SEM analyses, vide infra) of both xerogels showed the presence of the elements Si, O, and C, as expected. A determination of the chlorine content indicated minor contents of 1.15 wt% in case of **1A** and 0.89 wt% in case of **2A**. This corresponds well with the ^29^Si NMR studies in solution (see Section 2.2), where a complete substitution of all three (in case of CH_3_SiCl_3_) and all four (in case of SiCl_4_) chlorine atoms was observed for the pyridine catalyzed reactions.

The powder X-ray diffraction data indicated completely amorphous structures for xerogels **1A** and **2A**. In the vibrational spectra, all typical absorption bands for the characteristic C–H, C–C, C–O, Si–O, and Si–C valence and deformation vibrations were present (see materials and methods section).

**Figure 3:**
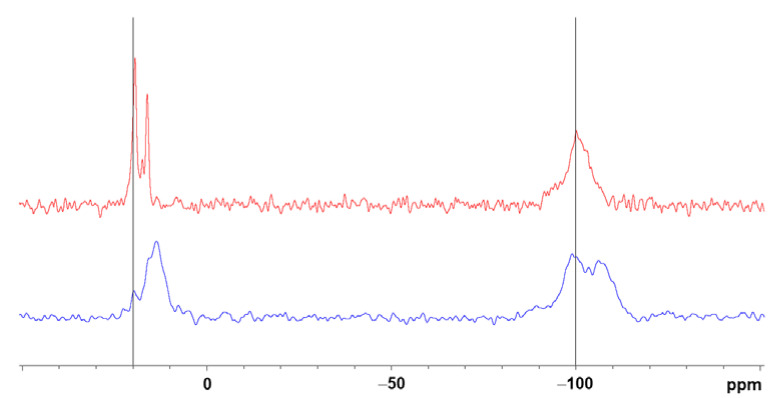
^29^Si cross-polarization magic angle spinning (CP MAS) NMR spectra of xerogel **1A** (obtained from **1**/SiCl_4_/pyridine/THF), top (red trace): xerogel prepared under argon; bottom (blue trace): same sample of xerogel after two days in contact with ambient air. (The vertical lines at *δ* = +20 ppm and −100 ppm aid enhanced visualization of the chemical shift differences).

**Figure 4:**
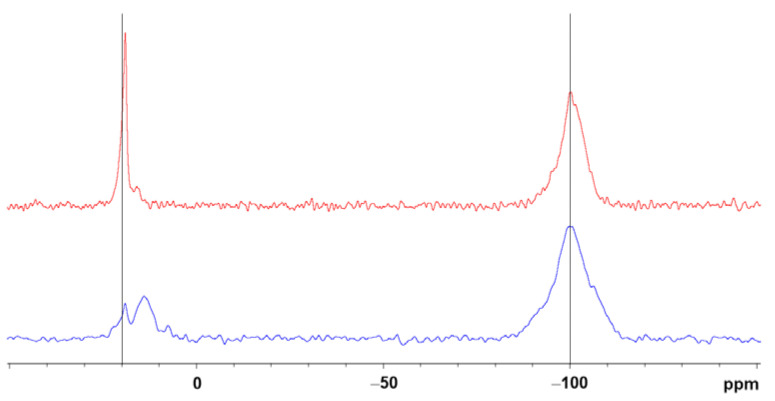
^29^Si CP MAS NMR spectra of xerogel **2A** (obtained from **2**/SiCl_4_/pyridine/THF), top (red trace): xerogel prepared under argon; bottom (blue trace): same sample of xerogel after two days in contact with ambient air. (The vertical lines at *δ* = +20 ppm and −100 ppm aid enhanced visualization of the chemical shift differences).

In Figure 3 and Figure 4, the solid state ^29^Si NMR spectra of both xerogels **1A** and **2A** are shown. Despite the fact that stoichiometric amounts of the starting materials SiCl_4_ and **1** or **2** were used, i.e., a molar ratio of 1:2, relatively strong signals for unreacted or partly reacted O–Si(CH_3_)_3_-groups in **1** (around 20 ppm), and some signals for potential side products with OSi(CH_3_)_3_-groups (signals in the range 10–16 ppm)), were found. The signals for the expected SiO_4_-moieties in the xerogels are clearly detectable (around −100 ppm). As the spectrum essentially consists of these two groups of signals, we conclude that moieties of partial substitution (ClSi(O-aryl)_3_, expected in the ^29^Si NMR shift range (−80 to −85 ppm) are almost absent. In Figure 3, it can be seen that after storing the powdered xerogel **1A** for two days in ambient air, new upfield shifted signals emerge for both groups of signals; we attribute them to (aryl-O)_3_Si–O–Si-groups. The spectrum of xerogel **1A** (in contrast to xerogel **2A**) already shows a sharp signal at 16.1 ppm before contact with air, which is attributed to adsorbed (CH_3_)_3_SiOH. After the contact with humidity from air, the unreacted Si(CH_3_)_3_)-groups in both xerogels were partially hydrolysed and bonded to the Si–O network of the xerogel. The backbone of xerogel **1A** is obviously more prone to hydrolysis when compared to that of xerogel **2A**, because after two days of storage with air contact, an upfield signal around −110 ppm is present in the spectrum of sample **1A** (Figure 3), but an analogous signal is less pronounced in the spectrum of **2A** upon the same treatment (Figure 4, bottom).

This higher sensitivity toward hydrolysis of **1A** may be caused by the fact that the biphenyl units provide more flexibility and more accessibility for water molecules due to the torsional freedom between both phenylene groups, while the naphthalene fragment is essentially rigid.

### 2.4. Morphological and Thermal Characterization of the Xerogels

**Figure 5:**
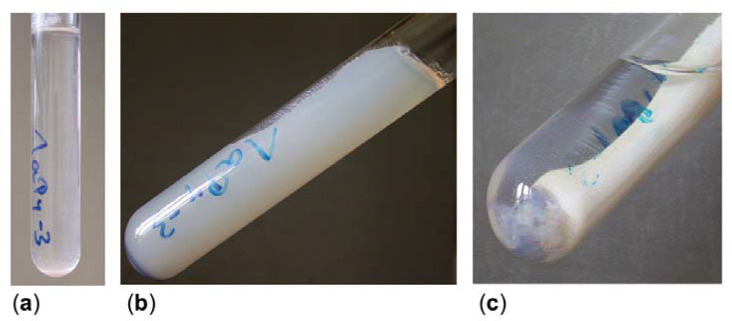
Reaction mixture **1aPy** and formation of gel **1A**: (**a**) at t = 0 h, (**b**) after one day gelation and partial syneresis already occurred, (**c**) aged for 21 days.

The phenomenology of the Sol–Gel transition observed for the hybrid Sol–Gel system presented here is very similar to typical oxide Sol–Gel processes: a homogenous and transparent liquid reaction mixture solidifies—after a certain reaction time of typically hours to days—with formation of a shape retaining monolithic gel body that is usually translucent for visible light. In Figure 5, the appearance of gels of the system **1aPy** is depicted.

**Figure 6:**
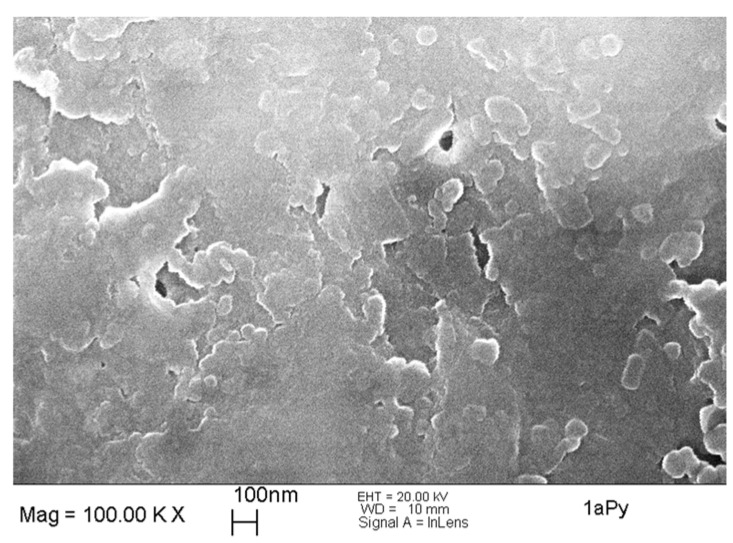
SEM image of xerogel **1A** (obtained from **1**/SiCl_4_/pyridine/THF).

The morphology of the xerogels was investigated using scanning electron microscopy (SEM). The image in Figure 6 shows that xerogel **1A** is constituted of nanostructured primary particles with diameters of less than 50 nm. These primary particles are very strongly agglomerated, fused, or even coalesced.

**Figure 7:**
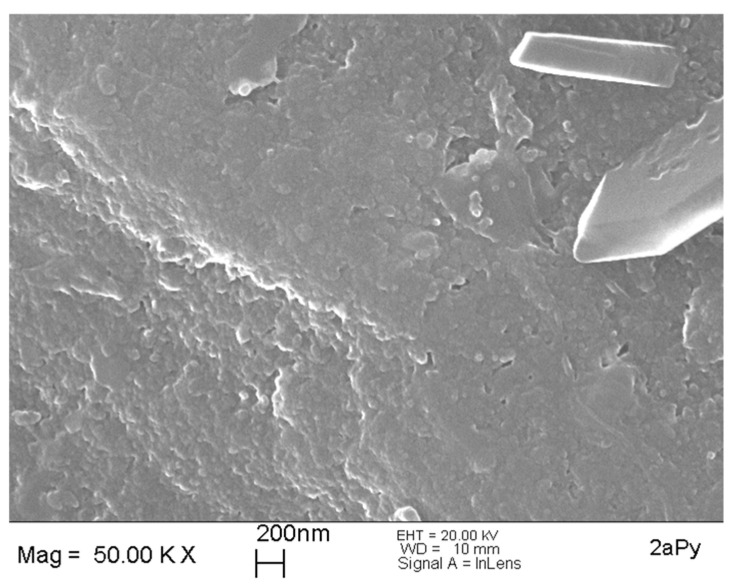
SEM image of xerogel **2A** (obtained from **2**/SiCl_4_/pyridine/THF). The composition and identity of the larger crystalline-appearing particle in the upper right corner of the image could not be resolved.

Xerogel **2A** is characterized by a very similar microstructure, with primary particle sizes in the range below 50 nm, as can be seen in Figure 7. The degree of agglomeration is very similar to that of xerogel **1A**. The particles are almost fully coalesced to a dense homogeneous body, with only very little inter-particle spaces and corresponding porosity.

Nitrogen adsorption measurements, analyzed according to the Brunauer–Emmett–Teller (BET) model, indicated that xerogel **2A** has a surface area of approximately 10 m^2^⋅g^−1^. This relatively low value corresponds very well with the SEM images, which show that most of the porosity and surface area (which was most likely present in the wet gels) strongly decreases during ageing and drying of the gels. This is also supported by the large linear shrinkage of 57% for xerogel **1A** and 59% for xerogel **2A**.

**Figure 8:**
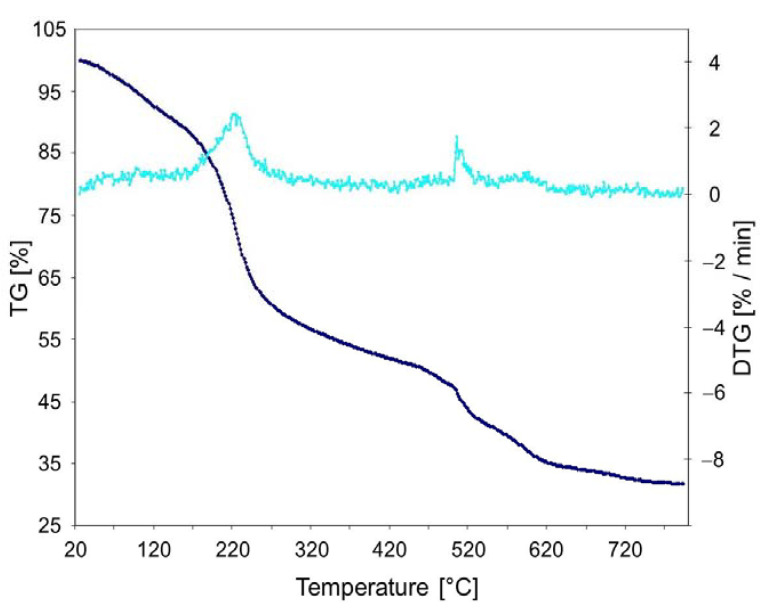
TG/DTG measurement of xerogel **1A** (obtained from **1**/SiCl_4_/pyridine/THF; TG trace dark blue, DTG trace light blue).

**Figure 9:**
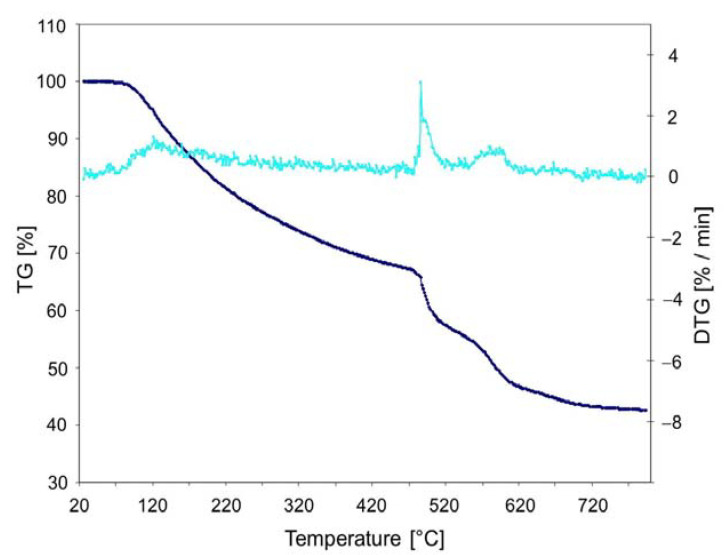
TG/DTG measurement of xerogel **2A** (obtained from **2**/SiCl_4_/pyridine/THF; TG trace dark blue, DTG trace light blue).

The thermal behavior of the xerogels was investigated under argon atmosphere applying a heating rate of 5 K⋅min^−1^ up to 800 °C. Further TG-FTIR studies were performed in the temperature range up to 750 °C, again with a heating rate of 5 K⋅min^−1^ under argon. The results of the measurements are shown in Figure 8 and Figure 9.

The TG/DTG curves of xerogels **1A** and **2A** are similar, showing three peaks in the comparable temperature ranges (**1A**: ca. 220 °C, 520 °C, and 600 °C; **2A**: ca. 150 °C, 520 °C, and 600 °C). For both xerogels, the mass loss below 250 °C can be attributed at least in part to the desorption of residual THF and pyridine. This was supported by FTIR spectra of the evolved gases. Desorption of volatile species containing –O–Si(CH_3_)_3_ groups and also condensation reactions of –O–Si(CH_3_)_3_ terminal groups also occurs. Hexamethyldisiloxane and trimethylsilanol were clearly detectable in the FTIR spectra. At temperatures around 500 °C and 600 °C, decomposition reactions of aromatic C–H and remaining trimethylsilyl units led to the formation of methane for both xerogels.

In the case of xerogel **1A**, the total mass loss in the temperature range from 20 °C to 800 °C is 68.2%; xerogel **2A** displays a lower mass loss of 57.3% in the same temperature range. Although the pyrolytic residues were not analyzed, it is expected that Si/C/O/(H) materials are formed at 800 °C. Thus, a slightly higher thermal stability and a higher ceramic yield is found for xerogel **2A**; this may be attributed to a higher degree of “graphitization” and, in turn, a higher thermal stability of the naphthalene moieties when compared to the biphenylene units.

## 3. Conclusions

Here we reported on a non-hydrolytic Sol–Gel system for “organically bridged” or “linearly expanded” silica and silsesquisiloxane hybrid materials. The synthesis is based on reactions of SiCl_4_ and CH_3_SiCl_3_ with rigid aromatic diols, which are two-fold trimethysilylated, namely compounds **1** and **2**. The driving force for these reactions is the formation of (CH_3_)_3_SiCl.

The reactions proceed smoothly at room temperature and are accelerated by the addition of catalytic amounts of pyridine. Most importantly, pyridine addition also causes a complete substitution of all three or four chlorine atoms in the stoichiometric reaction mixtures with CH_3_SiCl_3_ and SiCl_4_, respectively, even at room temperature.

A Sol–Gel transition is observed only in case of reactions of SiCl_4_ in the presence of pyridine. The gels exhibit strong shrinkage, which leads to rather dense, nearly non-porous xerogels **1A** and **2A**, both of which are hydrolytically sensitive.

The route to organic-inorganic hybrid polymers and gels presented here may be applied to many related systems. We expect that other chlorosilanes with at least three chlorine atoms as well as other element halides with similar reactivity may be combined in suitable inert solvents with trimethylsilylated di-, tri-, or polyols to form (CH_3_)_3_SiCl or (CH_3_)_3_SiX and more-or-less branched and cross-linked network structures. The products may be calcined to form amorphous organic-inorganic hybrids with tailorable elemental composition and properties.

The hydrolytic sensitivity of as prepared xerogels may not necessarily be a disadvantage. This property might be exploitable for the development of environmentally degradable materials or for a targeted release of incorporated components triggered by contact with water.

## 4. Materials and Methods

General Techniques and Chemicals. All manipulations were performed under an inert (Ar or N_2_) atmosphere using standard Schlenk techniques or in a glovebox. The solvents, tetrahydrofuran (THF), and *n*-hexane were dried by distillation from a sodium/benzophenone mixture. The remaining starting materials, i.e., 4,4′-dihydroxybiphenyl, 2,6-dihydroxynaphthalene, hexamethyldisilazane (HMDS), potassium tert-butanolate (KOtBu), as well as the chlorosilanes SiCl_4_ and CH_3_SiCl_3_, were used as received without further purification.

Synthesis of the starting materials. 4,4′-Bis(trimethylsiloxy)biphenyl (**1**): The compound was synthesized according to a procedure reported in the literature [65]. However, the brownish product was impure and had to be purified. This was performed by extracting 45.0 g (136 mmol) of the raw product with 50 mL boiling n-hexane. In the extract solution, white crystals of the product formed at room temperature. After filtration and drying under vacuum, 40.9 g (124 mmol) analytically pure **1** were obtained. Mp 64 °C. NMR: ^1^H (400 MHz, CDCl_3_) δ [ppm]: 7.42 (m, 4H, ^3^J_HH_ = 8.4 Hz, H2,2′), 6.88 (m, 4H, ^3^J_HH_ = 8.4 Hz, H3,3′), 0.31 (s, 18H, CH_3_); ^13^C (100 MHz, CDCl_3_) δ [ppm]: 154.3 (C4, 4′), 134.2 (C1,1′), 127.7 (C2, 2′), 120.2 C3,3′), 0.2 (CH_3_); ^29^Si (79.5 MHz, CDCl_3_) δ [ppm]: 19.7 (s). IR (KBr pellet), ν [cm^−1^]: 3035–2960 (w) [aromatic C–H valence vibration]; 2900 (w) [C–H valence vibration of CH_3_-groups]; 1500 (m), 1600 (m) [aromatic C=C valence vibration]; 1263–1248 (s) [C–O valence vibration]; 1260–1250 (s), 843–817 (s), 760–752 (m) [Si(CH_3_)_3_ valence vibration]; 1174 (w), 1102 (w), 936–919 (s) [Si–O–C(aryl) valence vibration]. Elemental analysis calcd (%) for C_18_H_26_O_2_Si_2_ (M = 330.56 g/mol): C 65.40, H 7.93, found: C 65.35, H 8.09.

2,6-Bis(trimethylsiloxy)naphthalene (2): In a round bottomed flask, 5.0 g (31 mmol) 2,6-dihydroxynaphthalene was mixed with 18 mL (86 mmol) of hexamethyldisilazane (HMDS) and a catalytic amount (~50 mg) of potassium tert-butanolate. The reaction mixture was heated under reflux with vigorous stirring for 1 h. The end of the reaction was indicated when the ammonia formation has ceased. Isolation of the product was accomplished using fractioned distillation. After removal of excessive HMDS, pure **2** was collected at 130–134 °C/1.3–2.6 × 10^−2^ Pa as a colorless liquid. Upon cooling to room temperature, the distillate formed a white crystalline solid. Yield: 9.2 g (30 mmol, 97%). Mp 109 °C. NMR: ^1^H (400 MHz, CDCl_3_) δ [ppm]: 7.58 (d, 2H, ^3^J_HH_ = 8.8 Hz, H^4,8^), 7.15 (d, 2H, ^4^J_HH_ = 1.8 Hz, H^1,5^), 7.03 (dd, 2H, ^3^J_HH_ = 8.8 Hz, ^4^J_HH_ = 1.8 Hz, H^3,7^), 0.3 (s, 18H, CH_3_); ^13^C (100 MHz, CDCl_3_) δ [ppm]: 151.5 (C2), 130.3 (Cq), 128.0 (C4), 122.3 (C3), 114.9 (C1), 0.3 (CH_3_); ^29^Si (79.5 MHz, CDCl_3_) δ [ppm]: 19.9 (s). IR (KBr pellet), ν [cm^−1^]: 3050 (w), 2955 (w) [aromatic C-H valence vibration]; 2900 (w) [C–H valence vibration of CH_3_-groups]; 1600 (m), 1500 (m) [aromatic C=C valence vibration]; 1390–1375 (m) [CH_3_-deformation vibration]; 1270–1250 (s), 1121 (m), 1160 (m) [C–O valence vibration]; 1261–1244 (s), 874–847 (vs), 811 (w), 761 (m) [Si(CH_3_)_3_ valence vibration]; 966–958 (m) [Si–O–C(aryl) valence vibration]. Elemental analysis calcd (%) for C_16_H_24_O_2_Si_2_ (M = 304.53 g/mol): C 63.11, H 7.94, found: C 62.85, H 8.18. A single-crystal X-ray structure analysis of **2** was reported earlier [66] and provided further detailed information on this compound.

Polymer and Gel Synthesis. The polymer synthesis was performed analogous to the preparation of s-triazine- and s-heptazine-based hybrid materials, as described in [54]. In a typical experiment, 1 mmol of **1** was dissolved in 5 mL of dry THF and 0.5 mmol of SiCl_4_ was added at room temperature in a Schlenk tube. For comparison, the same experiment was conducted with 0.1 mmol of pyridine added as a catalyst. The liquid homogeneous reaction mixtures were stored at room temperature or heated to 60 °C. For the synthesis of less cross-linked polymers, 0.33 mmol of methyltrichlorosilane and 0.5 mmol of **1** were dissolved in 4 ml of dry THF, in absence and presence of 0.05 mmol of pyridine, and stored at room temperature or heated to 60 °C. Syntheses of polymers derived from the naphthalene derivative **2** were conducted analogously. Further details are summarized in Table 2 and Table 3.

Gelation was observed only in the cases of the pyridine catalyzed experiments with SiCl_4_. After an aging period of 21 days at 60 °C, the gels were carefully dried in vacuum. The obtained xerogels **1A** and **2A** consisted of glass-like bulk xerogel bodies. Yield and linear shrinkage were determined followed by characterization with powder XRD, gas adsorption (BET), elemental analysis, simultaneous thermal analysis (TG/DTA), and IR and ^29^Si CP/MAS NMR spectroscopy:

**1A**: Yield = 173 mg of a white, solid xerogel. NMR (CP/MAS at 5 kHz) δ [ppm]: ^29^Si (79.5 MHz): −100.2 (broad, SiO_4_-units), further sharp signals at 19.4, 17.4 and 16.1 (O–Si(CH_3_)_3_ groups). IR (KBr-pellet), ν [cm^−1^]: 2960(vw) [aromatic C–H valence vibration]; 1600 (w), 1500 (w) [aromatic C=C valence vibration]; 1250 (m) [C–O valence vibration]; 1251 (m), 845 (w), 821 (w) [Si(CH_3_)_3_ valence vibration]; 1169 (w), 1107 (w), 936 (w), 913 (w) [Si-O-Ar valence vibration]. Elemental analysis calcd (%) for [C_24_H_16_O_4_Si]_n_ (M = 396.45 g/mol): Cl 0.0., Si 7.08, found: Cl 1.15, Si 8.79.

**2A**: Yield = 153 mg of a white and very rigid xerogel. NMR (CP/MAS at 5 kHz) δ [ppm]: ^29^Si (79.5 MHz): −100.1 (broad, SiO_4_-units), 19.0 (sharp, O–Si(CH_3_)_3_ groups). IR (KBr-pellet), ν [cm^−1^]: 3065 (vw), 2958 (vw) [aromatic C–H valence vibration]; 1600 (m), 1505 (w) [aromatic C=C valence vibration]; 1375 (m) [CH_3_-deformation vibration]; 1230 (m), 1119 (w), 1150 (w) [C–O valence vibration]; 870 (w), 802 (w), 750 (vw) [Si(CH_3_)_3_ valence vibration]; 983 (m) [Si–O–Ar valence vibration]. Elemental analysis calcd (%) for [C_20_H_12_O_4_Si]_n_ (M = 344.38 g/mol): Cl 0.0., Si 8.2, found: Cl 0.89, Si 10.8.

Methods of Characterization. All manipulations of gels, xerogels, and pyrolysis products were performed under inert gas using glove box and/or Schlenk techniques.

IR Spectra were recorded using the standard KBr pellet method. For the starting materials, a Nicolet 510 FTIR spectrometer was used. The IR investigation of the xerogels was performed on a Varian FTIR spectrometer.

Solution ^1^H, ^13^C and ^29^Si NMR spectra were recorded on a BRUKER DPX 400 instrument at room temperature (TMS as internal standard). The ^29^Si NMR spectra were measured using inverse gated decoupling (IGATED).

Solid State ^29^Si NMR data of the dried gels **1A** and **2A** were acquired on a BRUKER AvanceTM WB 400 MHz spectrometer with a resonance frequency of 79.51 MHz using magic-angle spinning (MAS) at 5 kHz in 7 mm ZrO_2_ rotors and cross polarization (CP) with 5 ms contact time. The chemical shift is referenced externally to octakis(trimethylsiloxy)octasilsesquioxane Q_8_M_8_ (the most upfield signal of its Q^4^ groups at δ_iso_ = −109 ppm) and reported relative to tetramethylsilane (TMS).

Powder X-ray Diffraction (XRD): The patterns of the xerogels were obtained with a Siemens D-5000 diffractometer with Cu K_α_ radiation (λ = 1.5418 Å).

Single-crystal X-ray diffraction analysis of **2***:* When the freshly vacuum-distilled 2,6-bis(trimethylsiloxy)naphthalene had solidified, a tiny piece of the solid was found to be suitable for X-ray structure analysis. X-ray diffraction data were collected on a BRUKER-NONIUS X8 APEXII-CCD diffractometer with Mo-K_α_-radiation (*λ* = 0.71073 Å). The structure was solved with direct methods and refined with full-matrix least-squares methods. All non-hydrogen atoms were refined anisotropically. Hydrogen atoms were placed in idealized positions and refined isotropically (riding model). Structure solution and refinement of *F*^2^ against all reflections were performed with SHELXS-97 and SHELXL-97 (G.M. Sheldrick, Universität Göttingen, Göttingen, Germany, 1986–1997).

2,6-bis(trimethylsiloxy)naphthalene: C_16_H_24_O_2_Si_2_, M_r_ = 304.53, colourless piece, 0.45 × 0.40 × 0.06 mm^3^, trigonal space group *R*-3, *a* = *b* = 21.0302(4), *c* = 10.5261(4) Å, *α* = *β* = 90°, *γ* = 120°, *V* = 4031.67(19) Å^3^, *Z* = 9, *ρ*_calcd._ = 1.129 g⋅cm^−3^, 2*θ*_max_ = 60°, *F*(000) = 1476, *μ* = 0.197 mm^−1^, no absorption correction, *T* = 203(2) K, 24,421 recorded reflections (−29 ≤ *h* ≤ 29, −29 ≤ *k* ≤ 27, −14 ≤ *l* ≤ 14), 2608 unique and 2026 observed reflections with *F*_o_ > 2 *σ*(*F*_o_), 91 parameters, *R*1 = 0.0401, *wR*2 = 0.1083 [*I* > 2*σ*(*I*)], *R*1 = 0.0578, *wR*2 = 0.1179 (all data), residual electron density (highest peak and deepest hole) 0.410 and −0.341 eÅ^−3^.

Crystallographic data (excluding structure factors) for the structure reported in this paper have been deposited earlier with the Cambridge Crystallographic Centre as private communication CCDC 622212. These data can be obtained free of charge from the Cambridge Crystallographic Data Centre via https://www.ccdc.cam.ac.uk/structures/ (accessed on 27 February 2023).

High Resolution Scanning Electron Microscopy (HR-SEM) was performed with an LEO1530 microscope equipped with an energy dispersive X-ray (EDX) system.

Nitrogen Adsorption Measurements (Micromeritics ASAP 2000) were carried out after desorption at 150 °C/~0.5 mbar for 12 h of samples (~0.5 g) which were ground in a mortar under inert conditions.

Thermal Analysis was first performed using a Seiko Instruments TG/DTA 22 under argon atmosphere applying a heating rate of 5K⋅min^−1^. Further TG-FTIR studies were performed on a Setaram Sensys TG-DSC coupled with a Varian FTIR instrument in the temperature range up to 750 °C with a heating rate of 5 K⋅min^−1^ under argon. Alumina crucibles were used.

Elemental Analyses of the starting materials **1** and **2** were carried out on a “Foss Heraeus CHN-O-Rapid” analyzer while the determination of chlorine and silicon contents in **1A** and **2A** was performed by the “Mikroanalytisches Labor Pascher” (Remagen-Bandorf, Germany).

## Figures and Tables

**Table 1:** Selected bond lengths and angles for compound **2**. Symmetry equivalent atoms are marked with an asterisk *.

Bond Lengths [Å]		Bond Angles [°]	
C1–C2	1.424(2)	C1*–C1–C2	119.2(2)
C2–C3	1.364(2)	C1–C2–C3	120.5(1)
C3–C4	1.415(2)	C2–C3–C4	120.0(1)
C4–C5	1.364(2)	C3–C4–C5	120.6(1)
C5–C1*	1.413(2)	C4–C5–C1*	120.7(1)
C1–C1*	1.413(2)	C5*–C1–C1*	119.0(2)
		C5*–C1–C2	121.9(1)

**Table 2:** Overview about the reactions of precursor **1** with SiCl_4_ and CH_3_SiCl_3_ in THF.

Reaction Mixture	n(1) [mmol]	THF [mL]	n(SiCl_4_) [mmol]	n(CH_3_SiCl_3_) [mmol]	n_Pyridine_ [mmol]	Observation
**1a**	1	5	0.5	-	-	no gelation after three weeks at 60 °C
**1aPy**	1	5	0.5	-	0.1	gelation after 1 day; strong syneresis in following weeks, linear shrinkage of about 57% for xerogel 1A
**1b**	0.5	4	-	0.33	-	no gelation after three weeks at 60 °C
**1bPy**	0.5	4	-	0.33	0.05	

**Table 3:** Overview about the reactions of precursor **2** with SiCl_4_ and CH_3_SiCl_3_ in THF.

Reaction Mixture	n(2) [mmol]	THF [mL]	n(SiCl_4_) [mmol]	n(CH_3_SiCl_3_) [mmol]	n_Pyridine_ [mmol]	Observations
**2a**	1	5	0.5	-	-	no gelation after three weeks at 60 °C
**2aPy**	1	5	0.5	-	0.1	gelation after 1 day; strong syneresis in following weeks, linear shrinkage of 59% for xerogel 2A
**2b**	0.5	4	-	0.33	-	no gelation after three weeks at 60 °C
**2bPy**	0.5	4	-	0.33	0.05	

## Data Availability

Not applicable.

## Supplementary Materials

The following supporting information can be downloaded at: https://www.mdpi.com/article/10.3390/gels9040291/s1, Figure S1: ^29^Si solution NMR spectrum of 1b (1 with CH_3_SiCl_3_) after 1 day at room temperature; Figure S2: ^29^Si solution NMR spectra of reaction mixture 1b (1/CH_3_SiCl_3_/THF), top: after 1 day; center: after 8 days; bottom: after 30 days at room temperature; Figure S3: ^29^Si solution NMR spectra of reaction mixture 2b (2/CH_3_SiCl_3_/THF), top: after 1 day; center: after 8 days; bottom: after 30 days at room temperature; Figure S4: ^29^Si solution NMR spectra of reaction mixture 2bPy (2/CH_3_SiCl_3_/pyridine/THF), top: after 9 h; center: after 6 days; bottom: after 27 days at room temperature; Figure S5: ^29^Si solution NMR spectra of reaction mixtures 1a (1/SiCl_4_/pyridine/THF) [top: after 9 h (with broad absorption band for the glas of the NMR tube); bottom: after 7 days]; Figure S6: ^29^Si solution NMR spectra of reaction mixtures 2a (2/SiCl_4_/pyridine/THF) [top: after 18 h (with broad absorption band for the glas of the NMR tube); bottom: after 7 days]; Figure S7: Molecular structure of 2 in the crystal (thermal displacement ellipsoids plotted at the 50% probability level, H-atoms are omitted for clarity). The bond C1–C1* of the molecule is located on a crystallographic center of inversion, the atoms of the asymmetric unit are labeled, the asterisk * indicates a symmetry equivalent position. Figure S8: Xerogel **1A** after drying at 60 °C in vacuum for several hours. Table S1: Bond lengths [Å] of compound 2 (in its crystal structure); Table S2: Bond angles [deg.] of compound 2 (in its crystal structure); Table S3: Torsion angles [deg.] of compound 2 (in its crystal structure).
