# Supplementary material for: A Non-Hydrolytic Sol–Gel Route to Organic-Inorganic Hybrid Polymers: Linearly Expanded Silica and Silsesquioxanes
Source: Gels. 2023 Apr 2;9(4):291. doi: 10.3390/gels9040291 (PMC10138140; doi:10.3390/gels9040291)
Supplement: Supplementary file 1 [file gels-09-00291-s001.zip › gels-2306118-supplementary.pdf]

Supplementary Material

A Non-Hydrolytic Sol-Gel Route to Organic-Inorganic Hybrid Polymers: Linearly Expanded Silica and Silsesquioxanes

Katrin Krupinski [1], Jörg Wagler [1], Erica Brendler [2] and Edwin Kroke [1,*]

[1] Technische Universität Bergakademie Freiberg (TUBAF), Department of Chemistry and Physics, Institute for Inorganic Chemistry, Leipziger Strasse 29, 09596 Freiberg, Germany
[2] Technische Universität Bergakademie Freiberg (TUBAF), Department of Chemistry and Physics, Institute for Analytical Chemistry, Leipziger Strasse 29, 09596 Freiberg, Germany
* Correspondence: edwin.kroke@chemie.tu-freiberg.de; Tel.: +49-3731-39-3174; Fax: -4058

Contents

Figure S1. ^{29}Si solution NMR spectrum of **1b** (**1** with CH_3SiCl_3) after 1 day at room temperature.

Figure S2. ^{29}Si solution NMR spectra of reaction mixture **1b** (**1**/CH_3SiCl_3/THF), top: after 1 day; center: after 8 days; bottom: after 30 days at room temperature.

Figure S3. ^{29}Si solution NMR spectra of reaction mixture **2b** (**2**/CH_3SiCl_3/THF), top: after 1 day; center: after 8 days; bottom: after 30 days at room temperature.

Figure S4. ^{29}Si solution NMR spectra of reaction mixture **2bPy** (**2**/CH_3SiCl_3/pyridine/THF), top: after 9 hours; center: after 6 days; bottom: after 27 days at room temperature.

Figure S5. ^{29}Si solution NMR spectra (glass signal removed by baseline correction) of reaction mixtures **1a** (**1**/$SiCl_4$/pyridine/THF) [top: full spectrum of (**a**) the mixture after 9 hours and (**a′**) after 7 days]. Magnified sections with signal assgnment are shown in (**b**) and (**c**) for spectrum (**a**), in (**b′**) and (**c′**) for spectrum (**a′**).

Figure S6. ^{29}Si solution NMR spectra (glass signal removed by baseline correction) of reaction mixtures **2a** (**2**/$SiCl_4$/pyridine/THF) [top: full spectrum of (**a**) the mixture after 18 hours and (**a′**) after 7 days]. Magnified sections with signal assgnment are shown in (**b**) and (**c**) for spectrum (**a**), in (**b′**) and (**c′**) for spectrum (**a′**).

Figure S7. Molecular structure of **2** in the crystal (thermal displacement ellipsoids plotted at the 50 % probability level, H-atoms are omitted for clarity). The bond C1–C1* of the molecule is located on a crystallographic center of inversion, the atoms of the asymmetric unit are labeled, the asterisk * indicates a symmetry equivalent position.

Table S1. Bond lengths [Å] of compound **2** (in its crystal structure).

Table S2. Bond angles [deg.] of compound **2** (in its crystal structure).

Table S3. Torsion angles [deg.] of compound **2** (in its crystal structure).

Figure S8. Xerogel **1A** after drying at 60 °C in vacuum for several hours.

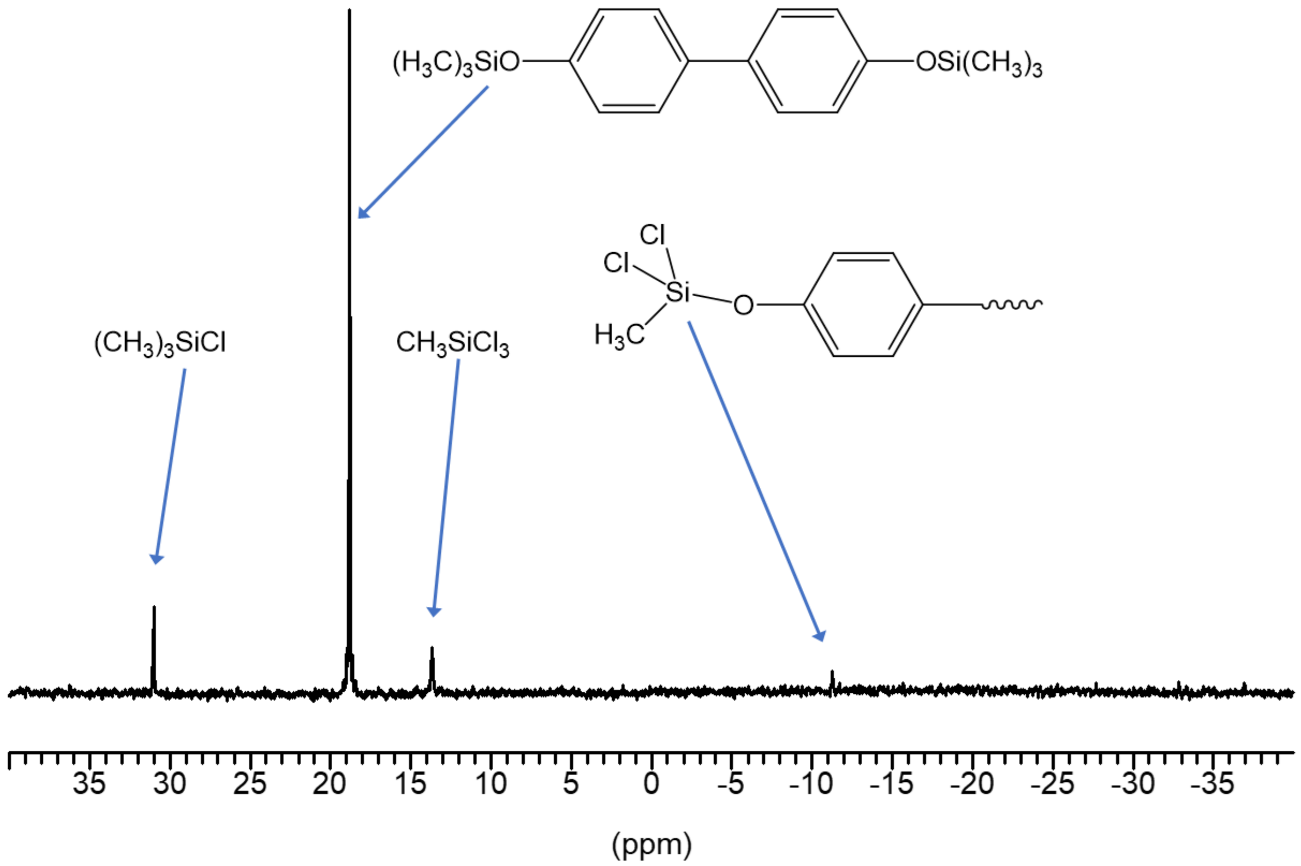

Figure S1. ^{29}Si solution NMR spectrum of **1b** (**1** with CH_3SiCl_3) after 1 day at room temperature.

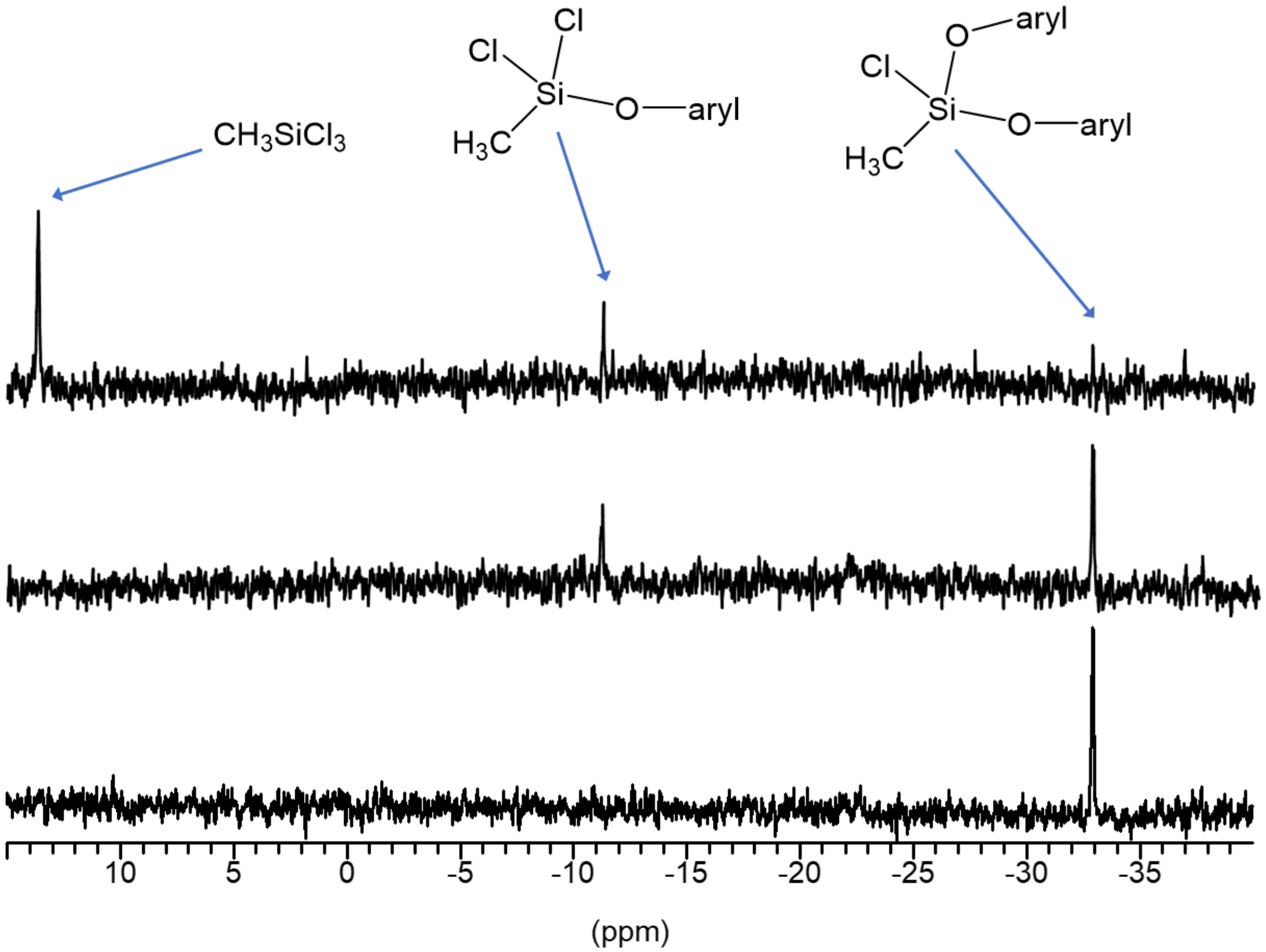

Figure S2. ^{29}Si solution NMR spectra of reaction mixture **1b** (**1**/CH_3SiCl_3/THF), top: after 1 day; center: after 8 days; bottom: after 30 days at room temperature.

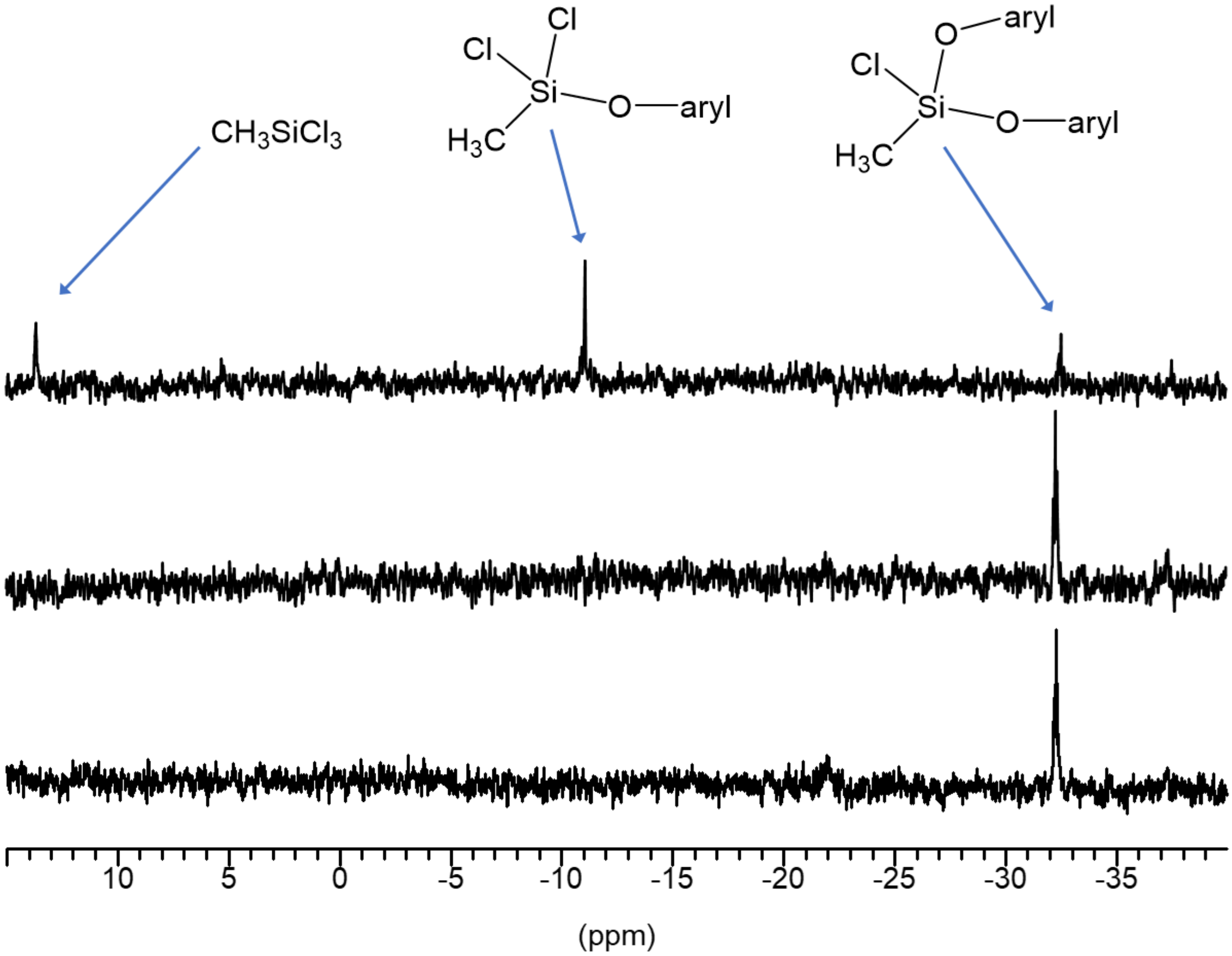

Figure S3. ^{29}Si solution NMR spectra of reaction mixture **2b** (**2**/CH_3SiCl_3/THF), top: after 1 day; center: after 8 days; bottom: after 30 days at room temperature.

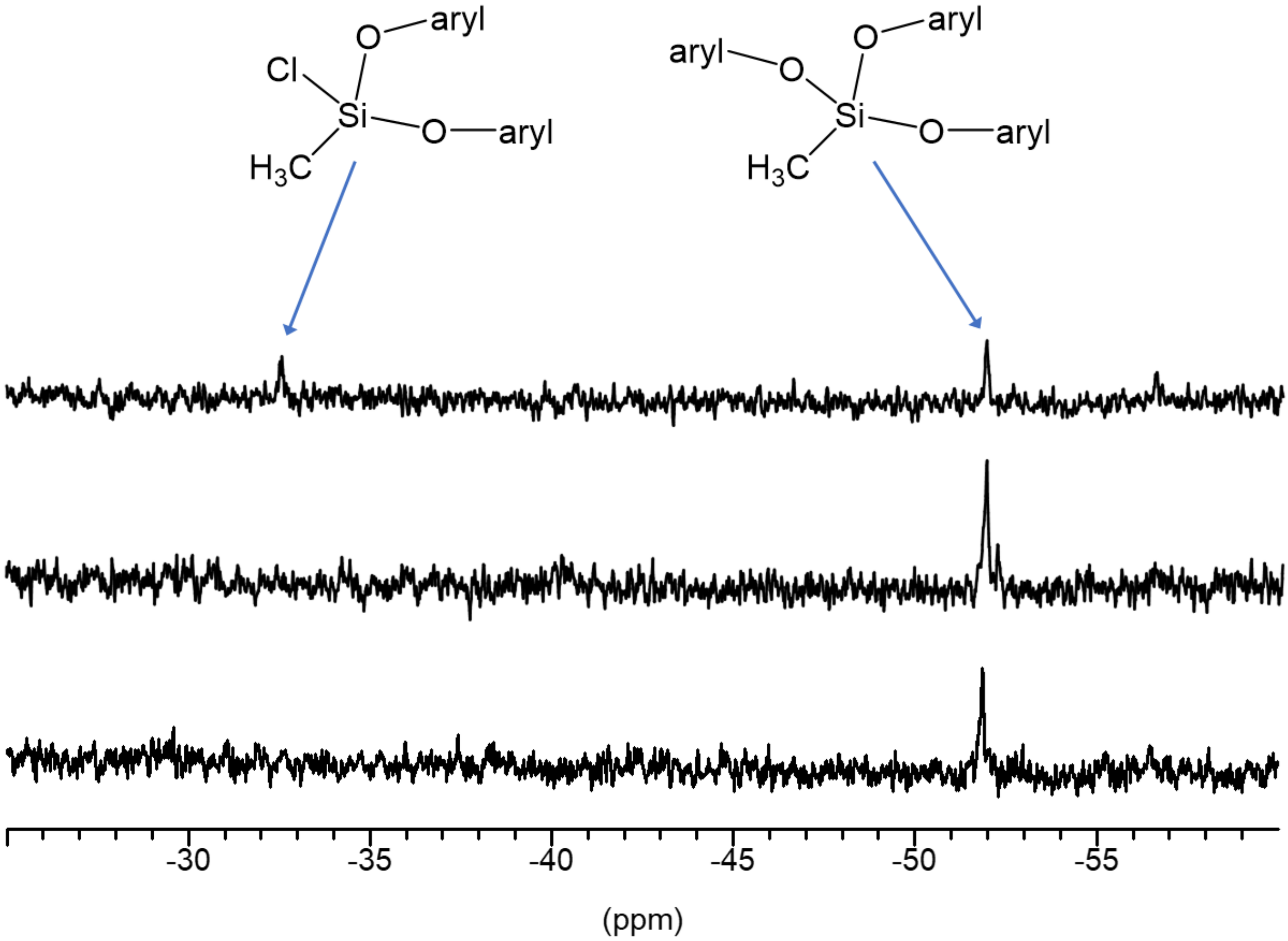

Figure S4. ^{29}Si solution NMR spectra of reaction mixture **2bPy** (**2**/CH_3SiCl_3/pyridine/THF), top: after 9 hours; center: after 6 days; bottom: after 27 days at room temperature.

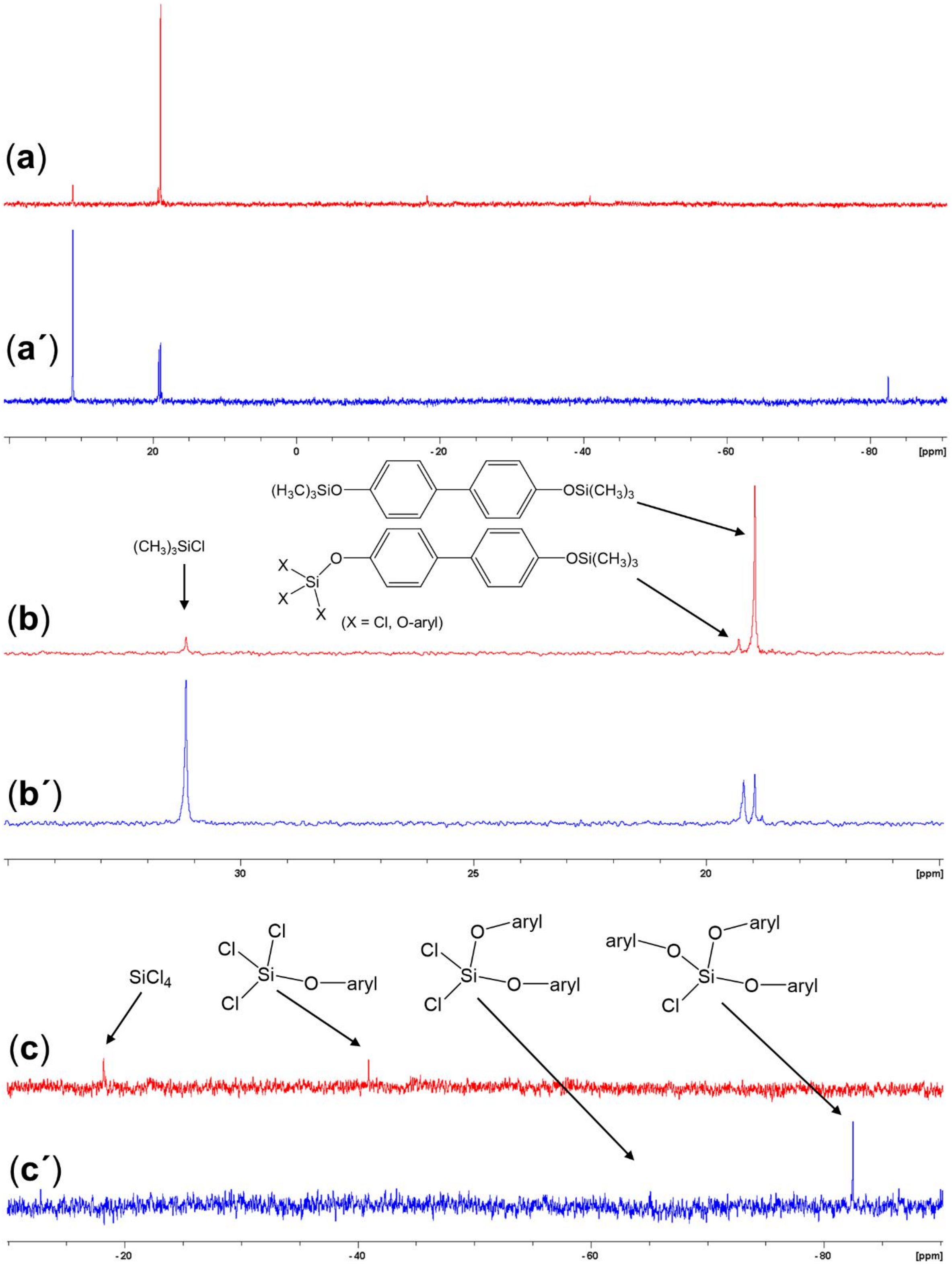

Figure S5. ^{29}Si solution NMR spectra (glass signal removed by baseline correction) of reaction mixtures **1a** (**1**/$SiCl_4$/pyridine/THF) [top: full spectrum of (**a**) the mixture after 9 hours and (**a′**) after 7 days]. Magnified sections with signal assgnment are shown in (**b**) and (**c**) for spectrum (**a**), in (**b′**) and (**c′**) for spectrum (**a′**).

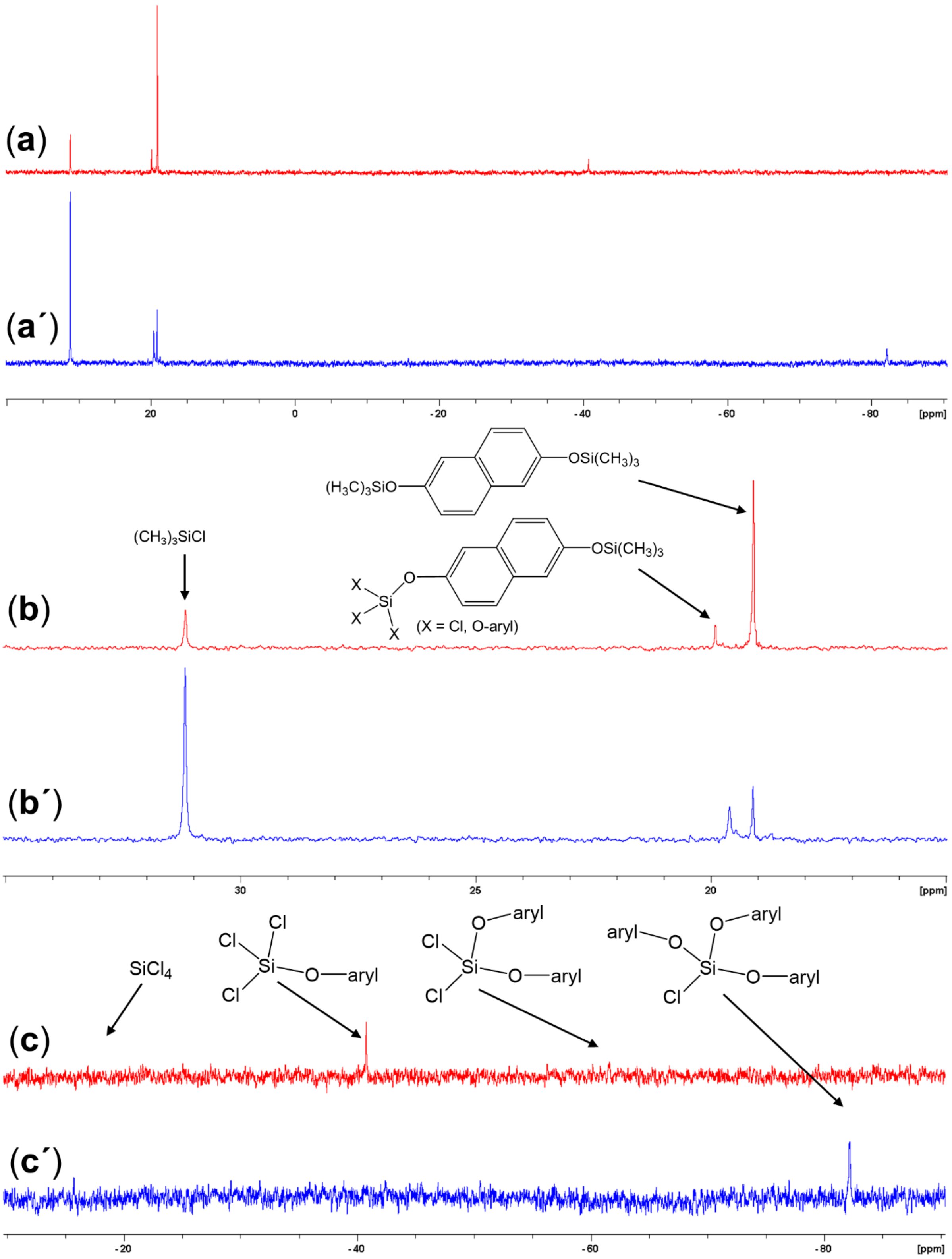

Figure S6. ^{29}Si solution NMR spectra (glass signal removed by baseline correction) of reaction mixtures **2a** (**2**/$SiCl_4$/pyridine/THF) [top: full spectrum of (**a**) the mixture after 18 hours and (**a′**) after 7 days]. Magnified sections with signal assgnment are shown in (**b**) and (**c**) for spectrum (**a**), in (**b′**) and (**c′**) for spectrum (**a′**).

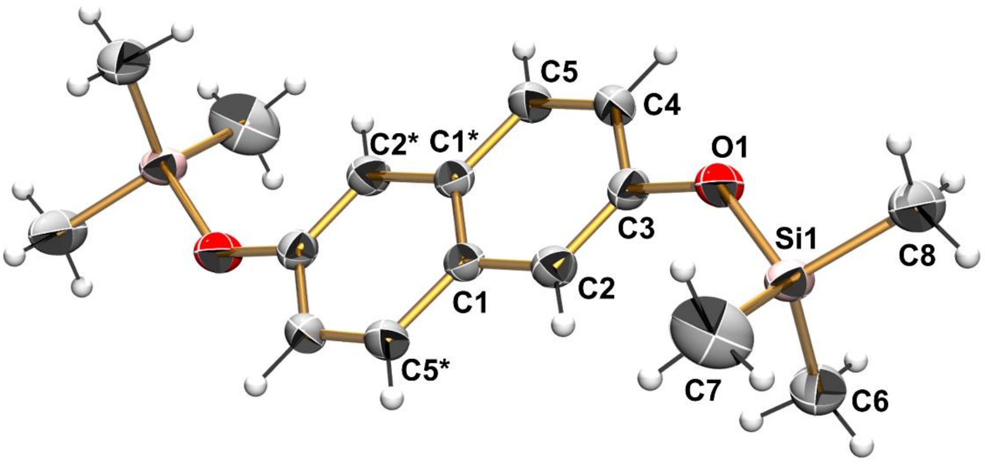

Figure S7. Molecular structure of **2** in the crystal (thermal displacement ellipsoids plotted at the 50 % probability level, H-atoms are omitted for clarity). The bond C1–C1* of the molecule is located on a crystallographic center of inversion, the atoms of the asymmetric unit are labeled, the asterisk * indicates a symmetry equivalent position.

Table S1. Bond lengths [Å] of compound **2** (in its crystal structure).

**Bond**	**Bond Length**	**Bond**	**Bond Length**
Si(1)-O(1)	1.6575(11)	C(1)-C(2)	1.4239(18)
Si(1)-C(6)	1.8436(18)	C(4)-C(5)	1.3635(19)
Si(1)-C(8)	1.8521(17)	C(4)-C(3)	1.4153(19)
Si(1)-C(7)	1.853(2)	C(3)-C(2)	1.3641(19)
C(1)-C(5)*	1.4127(18)	C(3)-O(1)	1.3707(16)
C(1)-C(1)*	1.413(2)		

Table S2. Bond angles [deg.] of compound **2** (in its crystal structure).

**Atoms**	**Bond Angle**	**Atoms**	**Bond Angle**
O(1)-Si(1)-C(6)	109.65(8)	C(1)*-C(1)-C(2)	119.17(15)
O(1)-Si(1)-C(8)	102.31(7)	C(5)-C(4)-C(3)	120.59(12)
C(6)-Si(1)-C(8)	112.00(10)	C(2)-C(3)-O(1)	124.00(13)
O(1)-Si(1)-C(7)	110.45(10)	C(2)-C(3)-C(4)	120.02(12)
C(6)-Si(1)-C(7)	111.02(11)	O(1)-C(3)-C(4)	115.96(12)
C(8)-Si(1)-C(7)	111.09(11)	C(3)-C(2)-C(1)	120.51(12)
C(5)*-C(1)-C(1)*	118.97(15)	C(3)-O(1)-Si(1)	132.31(9)
C(5)*-C(1)-C(2)	121.86(12)	C(4)-C(5)-C(1)*	120.74(12)

Table S3. Torsion angles [deg.] of compound **2** (in its crystal structure). Because of the crystallographically imposed inversion symmetry, torsion angles C(2)-C(1)-C(1)*-C(2)* and C(5)-C(1)-C(1)*-C(5)* are 180 deg. by definition.

**Atoms**	**Torsion Angle**	**Atoms**	**Torsion Angle**
C(5)-C(4)-C(3)-C(2)	-0.2(2)	C(2)-C(3)-O(1)-Si(1)	22.4(2)
C(5)-C(4)-C(3)-O(1)	-178.69(14)	C(4)-C(3)-O(1)-Si(1)	-159.10(12)
O(1)-C(3)-C(2)-C(1)	178.12(13)	C(6)-Si(1)-O(1)-C(3)	52.61(17)
C(4)-C(3)-C(2)-C(1)	-0.3(2)	C(8)-Si(1)-O(1)-C(3)	171.64(16)
C(5)*-C(1)-C(2)-C(3)	-179.78(13)	C(7)-Si(1)-O(1)-C(3)	-70.04(17)
C(1)*-C(1)-C(2)-C(3)	0.5(2)	C(3)-C(4)-C(5)-C(1)*	0.4(2)

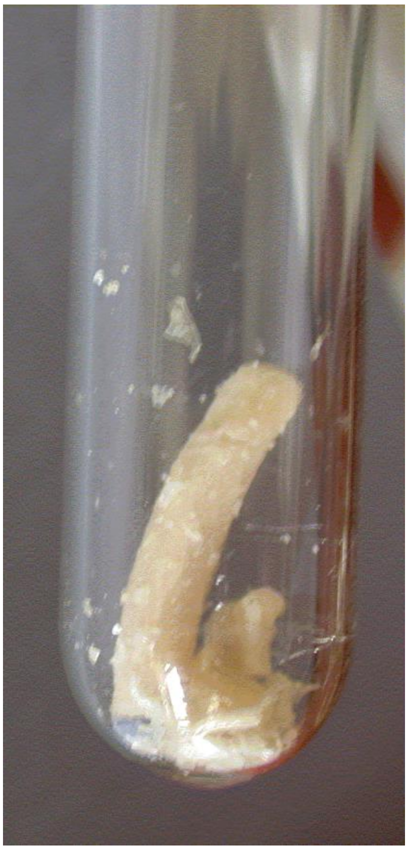

Figure S8. Xerogel **1A** after drying at 60 °C in vacuum for several hours.
